# Biodegradation of Triphenyl Phosphate by *Gordonia polyisoprenivorans* YC-XJ4: Environmental Adaptability and Bioremediation Potential

**DOI:** 10.3390/toxics14090815

**Published:** 2026-09-13

**Authors:** Xianjun Li, Xiao Ye, Junhuan Wang, Aikebaier Reheman

**Affiliations:** 1 Fujian Key Laboratory of Toxicant and Drug Toxicology, Medical College, Ningde Normal University, Ningde 352100, China; lixianjun1978@126.com (X.L.); yexiao_817@163.com (X.Y.); 2 School of Ocean Food and Biological Engineering, Jiangsu Ocean University, Lianyungang 222005, China

**Keywords:** organophosphate flame retardants, triphenyl phosphate, *Gordonia polyisoprenivorans*, biodegradation, bioremediation

## Abstract

Triphenyl phosphate (TPHP) is a widely used organophosphate flame retardant (OPFR) with concerning environmental persistence and toxicity. Microbial degradation is a promising removal strategy, but highly efficient degraders outside the *Sphingomonadaceae* family remain rarely reported. In this study, a highly efficient TPHP-degrading strain, designated YC-XJ4, was isolated from plastic-waste-contaminated soil and identified as *Gordonia polyisoprenivorans* through genomic analysis. The strain exhibited robust degradation activity over broad ranges of temperature (15–40 °C), pH (6.0–10.0), and salinity (0–2% NaCl), with a maximum average degradation velocity of 9.41 mg/(L·h) at an initial TPHP concentration of 300 mg/L and approximately 30% degradation retained at 500 mg/L. Substrate spectrum analysis showed that YC-XJ4 preferentially degraded aryl-OPFRs (TPHP, tricresyl phosphate, and 2-ethylhexyl diphenyl phosphate), whereas no degradation was detected for chlorinated or alkyl congeners. Whole-genome sequencing revealed the absence of known OPFR phosphotriesterase genes, suggesting that TPHP degradation in strain YC-XJ4 may be mediated by novel enzymes. In simulated bioremediation experiments conducted under natural outdoor conditions, the strain effectively removed TPHP from both soil and seawater. In soil, with a 5% (*v*/*v*) inoculum, the TPHP concentration decreased from 100 mg/kg to 4.66 mg/kg within 10 h (95.34% removal), while in seawater, 86.41% removal was achieved within 20 h at the same inoculum size. Collectively, *G. polyisoprenivorans* YC-XJ4 represents a promising non-*Sphingomonadaceae* degrader with strong environmental adaptability and practical bioremediation potential, providing a valuable candidate for both application and the discovery of novel degrading enzymes.

## 1. Introduction

Organophosphate flame retardants (OPFRs) are extensively added as plasticizers and flame retardants in a wide range of consumer products, including furniture, electronic and electrical equipment, transportation products, and building materials, leading to an ever-increasing release into the environment [1,2]. In certain occupational settings, OPFR contamination is particularly severe; for instance, the concentration of OPFRs in indoor dust from an electronic waste recycling site in Guangdong Province, China, has been reported to reach 0.218–2.90 mg/g [3]. Although the current exposure of the general population to OPFRs through daily-use products remains at a low-risk level, this exposure is expected to persist and even gradually increase over time [4]. Toxicological studies using human cell lines and zebrafish have demonstrated that OPFRs possess endocrine-disrupting potential and can disturb sex hormone homeostasis [5]. Furthermore, OPFRs have been shown to induce cardiotoxicity [6], developmental toxicity [7], neurotoxicity [8,9] and reproductive toxicity [10,11] in zebrafish, rats, and mice. The ubiquitous presence of OPFRs in the environment therefore poses a significant threat to both ecological safety and human health.

OPFRs can be eliminated from the environment through abiotic processes such as photolysis and chemical hydrolysis, as well as through biotic transformation mediated by terrestrial animals, plants, and microorganisms [12]. Among these, microbial degradation plays a particularly important role in the biotransformation of OPFRs. To date, a number of OPFR-degrading bacterial strains have been isolated from various environmental matrices, including river water, activated sludge, and landfill leachates. Many of the currently identified degrading strains belong to the family *Sphingomonadaceae*, exhibiting highly efficient OPFR-degrading capabilities. For instance, *Sphingomonas* sp. TDK1 completely hydrolyzed 6.5 mg/L tris(1,3-dichloro-2-propyl) phosphate (TDCPP) within 6 h, and *Sphingobium* sp. TCM1 completely hydrolyzed 5.7 mg/L tris(2-chloroethyl) phosphate (TCEP) within 3 h and 8.6 mg/L TDCPP within 6 h [13]. Recently, two *Sphingobium* strains capable of efficiently degrading tri-n-butyl phosphate (TNBP) were reported: *Sphingobium* sp. RSMS completely degraded 7980 mg/L TNBP within 3 days [14], and another *Sphingobium* sp. hydrolyzed 90% of TNBP (100 mg/L) within 63 h via sequential P–O bond cleavage [15]. Strains from this family can also efficiently degrade aryl-OPFRs. For example, *Sphingobium yanoikuyae* YC-XJ2 degraded over 99.0% of triphenyl phosphate (TPHP, 100 mg/L) within 24 h [16], and *Sphingopyxis terrae* subsp. *terrae* YC-JH3 hydrolyzed 87.7% of TPHP (100 mg/L) within 24 h [17,18].

OPFR-degrading strains belonging to other genera, including *Roseobacter*, *Brevibacillus*, and *Rhodococcus*, have also been documented; however, their degradation capabilities are generally limited, typically at least one order of magnitude lower than the above-mentioned strains. For instance, *Roseobacter* strain YS-57 required 30 h and 80 h to completely degrade TPHP and tricresyl phosphate (TCRP) at 0.7 mg/L, respectively [19]. *Brevibacillus brevis* was reported to degrade low concentrations (1 mg/L) of aryl-OPFRs, hydrolyzing 92.1% of TPHP, and 82.91%, 93.91%, and 53.92% of *m*-TCRP, *p*-TCRP, and *o*-TCRP, respectively, within 5 days [20,21]. *Rhodococcus pyridinivorans* YC-JH2 hydrolyzed only 37.36% of TPHP (50 mg/L) after 7 days of incubation [17,18]. Nevertheless, exceptions of highly efficient OPFR-degrading strains outside the *Sphingomonadaceae* family do exist. A notable example is *Rhodococcus pyridinivorans* YC-MTN, which achieved 99.24% degradation of TPHP (100 mg/L), 96.08% of TCRP, and 77.19% of 2-ethylhexyl diphenyl phosphate (EHDPP) within 72 h [22], demonstrating that certain non-*Sphingomonadaceae* members can also possess potent TPHP-degrading abilities.

Despite the growing number of OPFR-degrading bacteria isolated, the majority of studies have focused on strain isolation, degradation characteristics under laboratory conditions, and metabolic pathway elucidation, whereas the evaluation of their actual bioremediation potential in contaminated environmental matrices remains largely underexplored. This gap hinders the translation of microbial resources into practical cleanup strategies.

In recent years, *Gordonia polyisoprenivorans* has been increasingly recognized for its ability to degrade a variety of organic pollutants. For instance, *G. polyisoprenivorans* 135 was reported to degrade polycyclic aromatic hydrocarbons (PAHs) such as naphthalene and pyrene [23], while another isolate was found to effectively degrade carbamazepine, a persistent pharmaceutical contaminant [24]. Additionally, strains of this species have been shown to degrade rubber [25], polypropylene and polystyrene (*G. polyisoprenivorans* B253) [26], and various aromatic compounds, demonstrating its versatile catabolic potential and making it a promising candidate for environmental bioremediation. To the best of our knowledge, however, no studies have yet reported the degradation of OPFRs by *Gordonia* species, including *G. polyisoprenivorans*.

In the present study, a highly efficient TPHP-degrading strain, *Gordonia polyisoprenivorans* YC-XJ4, was isolated from plastic-waste-contaminated soil. The degradation capability of YC-XJ4 was systematically characterized under various environmental conditions (temperature, pH, salinity, metal ions, and surfactants), its substrate spectrum towards different OPFRs was determined, and its potential for remediating TPHP-contaminated soil and seawater was assessed through simulated bioremediation experiments. To our knowledge, this is one of the few reports of a *Gordonia* strain capable of efficiently degrading TPHP, and it provides both a promising bacterial candidate and fundamental insights for the bioremediation of OPFR-polluted environments.

## 2. Materials and Methods

### 2.1. Chemicals and Media

All chemicals and reagents were purchased from Shanghai Macklin Biochemical Co., Ltd. (Shanghai, China), including standards of OPFRs (TPHP, TCRP, EHDPP, TNBP, TCEP, tris(1-chloro-2-propyl) phosphate (TCPP), TDCPP), organic solvents of analytical grade or chromatographic grade (acetone, dichloromethane, methanol, acetonitrile, etc.), as well as inorganic salts and nutrient substances for medium preparation. Stock solutions of OPFRs were prepared in acetone at a concentration of 20,000 mg/L and stored at 4 °C until use.

The mineral salt medium was used to characterize the degradation capability of the degrading strain, while the nutrient medium was used to prepare the seed culture. The formulations are as follows:

Trace element medium (TEM): (NH_4_)_2_SO_4_ (2.0 g/L), Na_2_HPO_4_·12H_2_O (1.5 g/L), KH_2_PO_4_ (1.5 g/L), MgSO_4_·7H_2_O (0.2 g/L), CaCl·2H_2_O (0.01 g/L) and Trace Element Solution (TES) (100 µL/L), which contains MnSO_4_·2H_2_O (14.3 g/L), CoSO_4_·7H_2_O(1.2 g/L), CuSO_4_·5H_2_O (0.3 g/L), Na_2_MoO_4_·2H_2_O (0.2 g/L), Na_2_WO_4_·2H_2_O(2.3 g/L), FeSO_4_·7H_2_O (50 g/L) and ZnSO_4_·7H_2_O (2.2 g/L);

Luria–Bertani (LB): peptone (10 g/L), yeast (5 g/L), and NaCl (10 g/L).

For the solid medium, 1.6 g of agar powder was added per 100 mL of liquid medium. All media were sterilized by autoclaving at 121 °C for 20 min. Unless otherwise specified, the pH of the culture medium was 7.0. OPFRs were supplied to the trace element medium as the sole carbon source; it was experimentally verified that the test bacteria were unable to utilize acetone as a carbon source for cell growth. To ensure rigor, after the OPFRs stock solution was added to the TEM, the medium was placed in Laminar Flow Cabinet for at least 1 h to allow the solvent acetone to evaporate naturally.

### 2.2. Enrichment, Isolation, and Purification of TPHP-Degrading Bacteria

Soil samples collected from a plastic-waste-contaminated area were inoculated into 100 mL of TEM containing 50 mg/L TPHP and incubated at 30 °C and 180 rpm. Every 7 days, 10% (*v*/*v*) of the culture was transferred into fresh mineral salt medium with the TPHP concentration increased by 50 mg/L at each transfer. This procedure was repeated six times, leading to a final TPHP concentration of 300 mg/L. The enriched culture was then streaked onto mineral salt agar plates supplemented with 100 mg/L TPHP and incubated statically at 30 °C for 4 days. Single colonies were picked, streaked, and repeatedly purified until pure isolates were obtained. A purified strain with good growth, stable passage, and high TPHP-degrading capability was preserved and designated as YC-XJ4. Cell morphology of strain YC-XJ4 was observed by negative staining and transmission electron microscopy using a JEOL JEM-2100Plus (JEOL, Tokyo, Japan) at 200 kV.

### 2.3. Degradation Assays of Strain YC-XJ4

#### 2.3.1. Preparation of Seed Culture

Strain YC-XJ4 was inoculated into liquid LB medium for activation and cultured to the late logarithmic phase (*OD*_600_ = 0.7). The cells were harvested by centrifugation (5000× *g*, 5 min), washed three times with fresh TEM liquid medium, and resuspended in TEM to an *OD*_600_ of 0.7. This suspension was used as the seed culture.

#### 2.3.2. Degradation Experiments Under Different Environmental Conditions

Unless otherwise stated, all degradation assays were conducted in TEM liquid medium (10 mL) with an inoculum size of 1% (*v*/*v*) of the seed culture, an initial TPHP concentration of 200 mg/L, and incubation at 30 °C with shaking at 180 rpm in the dark. Abiotic controls (uninoculated medium containing the same TPHP concentration) were run in parallel for each condition; all treatments and controls were performed in triplicate. Residual TPHP was quantified after the indicated incubation periods.

Temperature effect was evaluated at 15, 20, 25, 30, 35, 40, and 45 °C for 24 h under pH 7.0. For pH experiments, the medium was adjusted to pH values ranging from 3 to 10, and incubation was carried out at 30 °C for 16 h. Salinity tolerance was assessed by adding NaCl to final concentrations of 0–4% (*w*/*v*) and incubating at 30 °C for 16 h. Metal ion effects were examined at 30 °C and pH 7.0 by supplementing the medium with individual metal ions (Pb^2+^, Mn^2+^, Hg^2+^, Cu^2+^, Ni^2+^, Co^2+^, Zn^2+^ and Cd^2+^) at 0.1 mM and 1 mM. Cultures were incubated for 16 h. Surfactant tolerance was tested at 30 °C and pH 7.0 using sodium dodecyl sulfate (SDS), ethylenediaminetetraacetic acid (EDTA), Tween 20, Tween 80, and Triton X-100 at concentrations of 0.1% and 1% (*w*/*v*); incubation lasted 12 h. To determine the effect of initial substrate concentration, TPHP was added at 100, 200, 300, 400, and 500 mg/L, and cultures were incubated at 30 °C and pH 7.0; samples were taken after 12 h and 24 h.

#### 2.3.3. Degradation Kinetics and Growth Curve of Strain YC-XJ4 on TPHP

To evaluate the degradation kinetics of TPHP and the concomitant growth of strain YC-XJ4, the seed culture was inoculated at 1% (*v*/*v*) into 10 mL of TEM (pH 7.0) containing 100 mg/L TPHP. Uninoculated TEM supplemented with 100 mg/L TPHP served as the abiotic control. All treatments and controls were performed in triplicate. The cultures were incubated at 30 °C with shaking at 180 rpm in the dark for 36 h. Samples were collected at 8 h intervals to determine the residual TPHP concentration and cell growth by measuring the optical density at 600 nm (*OD*_600_) using a Thermo spectroic 200 UV-Vis spectrophotometer (Thermo Fisher Scientific, Waltham, MA, USA).

#### 2.3.4. Substrate Spectrum Assay

The substrate spectrum of strain YC-XJ4 was determined at 30 °C and pH 7.0. The seed culture was inoculated into 10 mL of TEM at a ratio of 1% (*v*/*v*). Stock solutions of TPHP, TCRP, EHDPP, TNBP, TCEP, TCPP, and TDCPP were individually added to a final concentration of 200 mg/L. Cultures were incubated in a shaking incubator at 180 rpm for 24 h in the dark. Abiotic controls (uninoculated medium containing each OPFR at 200 mg/L) were run in parallel, and all treatments and controls were performed in triplicate.

#### 2.3.5. Extraction of OPFRs from Cultures

After incubation, the residual OPFRs were extracted by adding an equal volume of dichloromethane to the culture in a 50 mL conical flask. The mixture was shaken vigorously for 1 min and, after complete phase separation, 800 μL of the organic phase was transferred to a 1.5 mL microcentrifuge tube. The extract was evaporated to dryness in a fume hood, redissolved in 800 μL of methanol, vortexed for 1 min, and filtered through a 0.22 μm membrane filter prior to chromatographic analysis. TPHP, TCRP, and EHDPP were quantified by high-performance liquid chromatography (HPLC), while TNBP, TCEP, TCPP, and TDCPP were analyzed using a gas chromatography (GC). The extraction rate of all OPFRs in the TEM was above 98%.

### 2.4. Soil and Seawater Bioremediation Simulation Experiments

#### 2.4.1. Soil Bioremediation Experiment

Soil was collected from the campus garden of Ningde Normal University, sieved through a 40-mesh screen to remove coarse particles and stones. A portion of the soil was sterilized by autoclaving at 121 °C for 30 min and used for the inoculated treatments, while the remaining unsterilized soil was used for the control. For each treatment, 10 g of soil (sterilized or unsterilized according to the group) was placed in a 50 mL centrifuge tube. The experiment comprised a control (CK) and two inoculation ratios (1% and 5%, *v*/*v*). The CK consisted of unsterilized soil spiked with TPHP but not inoculated, whereas the treatment groups consisted of sterilized soil spiked with TPHP and inoculated with YC-XJ4 at 1% or 5% (*v*/*v*). TPHP was added to a final concentration of 100 mg/kg, and the total volume of TPHP stock solution plus TEM was adjusted to 5 mL per sample. Detailed information can be found in Table S1. The mixtures were vortexed thoroughly and incubated outdoors under natural conditions for 20 h. At 5 h intervals, the 10 g soil sample was extracted to determine the residual TPHP concentration. TPHP removal rate at each time point was calculated based on the initial spiked concentration (100 mg/kg), rather than the residual concentration measured in the control after 20 h of incubation; the latter was instead used to evaluate the removal contribution by indigenous soil microorganisms. All treatments and controls were performed in triplicate.

Each soil sample (10 g) was extracted with 20 mL of methanol by shaking vigorously for 2 h, followed by standing at 4 °C for 1 h. The mixture was centrifuged, and the methanol supernatant was collected in a 50 mL centrifuge tube. The extraction was repeated once, and the combined methanol extracts (approximately 40 mL) were evaporated to dryness in a fume hood. The residue was redissolved in 500 μL of acetonitrile, filtered through a 0.22 μm organic-phase membrane filter, and analyzed by HPLC. The extraction rate of TPHP from the soil was approximately 95%.

#### 2.4.2. Seawater Bioremediation Experiment

Coastal seawater was collected near the university. A portion of the seawater was filtered through a 0.22 μm membrane filter to remove indigenous microorganisms, and used as the matrix for the inoculated treatments, while the remaining unfiltered seawater was used for the control. For each treatment, 30 mL of seawater (filtered or unfiltered according to the group) was placed in a container. The experiment comprised a control (CK) and two inoculation ratios (1% and 5%, *v*/*v*). The CK consisted of unfiltered seawater spiked with TPHP but not inoculated, whereas the treatment groups consisted of filtered seawater spiked with TPHP and inoculated with YC-XJ4 at 1% or 5% (*v*/*v*). TPHP was added to a final concentration of 100 mg/L, and the total volume of TEM plus inoculum was brought to 10 mL per sample. Detailed information can be found in Table S2. The mixtures were vortexed for 1 min and incubated unsealed outdoors under natural conditions. Samples were taken at 5, 10, 15, and 20 h, extracted, and the TPHP concentration was determined. TPHP removal rates at each time point were calculated based on the initial spiked concentration (100 mg/L) rather than the residual concentration measured in the control after 20 h of incubation; the latter was instead used to evaluate the removal contribution by indigenous seawater microorganisms. Each group was set up in triplicate.

Each seawater sample was extracted with 10 mL of dichloromethane. After shaking, 600 μL of the lower organic phase was transferred to a 1.5 mL microcentrifuge tube, evaporated to dryness in a fume hood, and redissolved in 600 μL of acetone. The solution was filtered through a 0.22 μm membrane filter and analyzed by HPLC. The extraction rate of TPHP from seawater was around 98%.

### 2.5. Chromatographic Detection of OPFRs

TPHP, TCRP, and EHDPP were quantified by high-performance liquid chromatography (Thermo Scientific Ultimate 3000, Thermo Fisher Scientific, Waltham, MA, USA) with a mobile phase consisting of methanol and water (80:20, *v*/*v*) at a flow rate of 0.8 mL/min. The injection volume was 5 μL, the column temperature was maintained at 30 °C, and the detection wavelength was set at 205 nm. The total run time was 20 min. Under these conditions, the retention times were 16.73 min for TPHP, 8.44 min for TCRP, and 9.78 min for EHDPP.

TNBP, TCEP, TCPP, and TDCPP were quantified by gas chromatography (Thermo Scientific Trace 1310, MA, USA) equipped with an electron capture detector (ECD) set at 300 °C. The injection volume was 5 μL and N_2_ was used as the carrier gas at a flow rate of 1.51 mL/min. The oven temperature was programmed from 160 °C to 280 °C at 10 °C/min and held at 280 °C for 4 min. The detector make-up gases were hydrogen (40 mL/min) and air (400 mL/min). Under these conditions, the retention times were 4.67 min for TNBP, 5.05 min for TCEP, 5.33 min for TCPP, and 9.74 min for TDCPP.

Standard calibration curves were constructed for each compound using five concentration levels (5, 10, 20, 50, and 100 mg/L) prepared in methanol, and the peak area was used for quantification. The calibration curves for all seven OPFRs exhibited good linearity, with correlation coefficients (R^2^) greater than 0.99 (see Figures S1 and S2).

### 2.6. Accession Numbers and Homologous Sequence Analysis

The strain YC-XJ4 was subjected to whole-genome sequencing using a combination of Illumina (second-generation) and PacBio (third-generation) sequencing platforms by Majorbio (Shanghai, China), with each platform providing at least 100× coverage. The hybrid assembly strategy yielded a complete, closed genome, minimizing the loss of small plasmids (<15 kb) and ensuring the recovery of the complete genomic information including plasmids. The complete genome sequence has been deposited in GenBank under accession number GCA_059511865.1. Pairwise genome comparisons were performed using the Type (Strain) Genome Server (TYGS) to calculate digital DNA-DNA hybridization (dDDH) values, applying the recommended formula *d*_4_ and the established 70% species delineation threshold [27,28,29]. Further, Average Nucleotide Identity (ANI) was performed using online ANI calculator (https://www.ezbiocloud.net/tools/ani, accessed on 22 August 2026) [30]. To investigate the presence of OPFR-specific phosphotriesterase (PTE) homologs in the genome of strain YC-XJ4, a homologous sequence analysis was performed. The amino acid sequences of four previously characterized OPFR-specific PTEs (*Sm*-PTE(BAP16424.1), *Sb*-PTE (BAP16425.1), *Sy*-PTE (QNL13421.1) and *St*-PTE (QXF14330.1)) were retrieved from the GenBank database. These sequences were used as queries to search against the complete genome sequence of strain YC-XJ4 using BLASTP (Basic Local Alignment Search Tool for proteins) via the NCBI BLAST server (https://blast.ncbi.nlm.nih.gov/Blast.cgi), accessed on 5 May 2026.

### 2.7. Data Processing

All degradation experiments were performed in triplicate, and the results are presented as the mean ± standard deviation (SD). Statistical analysis was carried out using SPSS 19 software. One-way analysis of variance (ANOVA) followed by the least significant difference (LSD) test was used to assess the significance of differences among treatments. A *p* < 0.05 was considered statistically significant. Graphs were generated using GraphPad Prism 9.

## 3. Results

### 3.1. Isolation and Identification of Strain YC-XJ4

**Figure 1:**
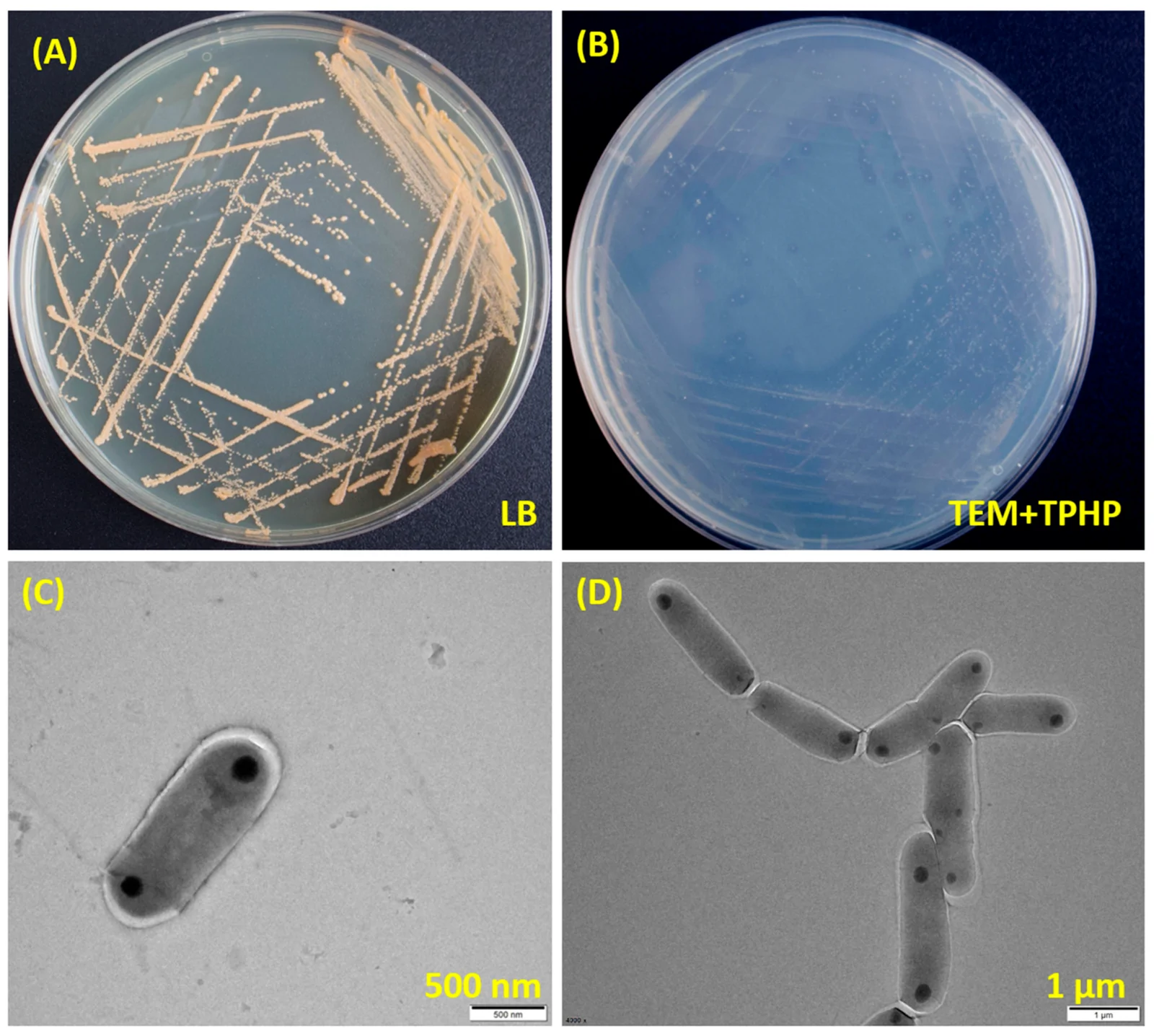
Morphological characteristics of strain YC-XJ4. Photographs of the strain on solid LB (**A**) and TEM (**B**) supplemented with TPHP (100 mg/L), showing transparent hydrolysis zones surrounding the colonies. (**C**,**D**) The morphological form of the strain under the transmission electron microscope.

After multiple rounds of enrichment and purification, a TPHP-degrading bacterium, designated YC-XJ4, was isolated from soil contaminated with plastic waste. On LB agar plates, colonies of strain YC-XJ4 were milky yellow, circular, and smooth-surfaced (Figure 1A). On TEM agar plates containing 100 mg/L TPHP, colonies appeared white, spherical, and smooth, with distinct clear zones of degradation (Figure 1B). Transmission electron microscopy revealed that cells of strain YC-XJ4 were short rod-shaped, approximately 2 μm in length, with a smooth surface and no flagella (Figure 1C). The cells contained two electron-dense intracellular inclusions, likely corresponding to storage granules such as polyphosphate bodies, and were surrounded by abundant extracellular secretions. They often adhered end-to-end to form long filaments or branched structures, or aggregated into clumps (Figure 1D).

**Figure 2:**
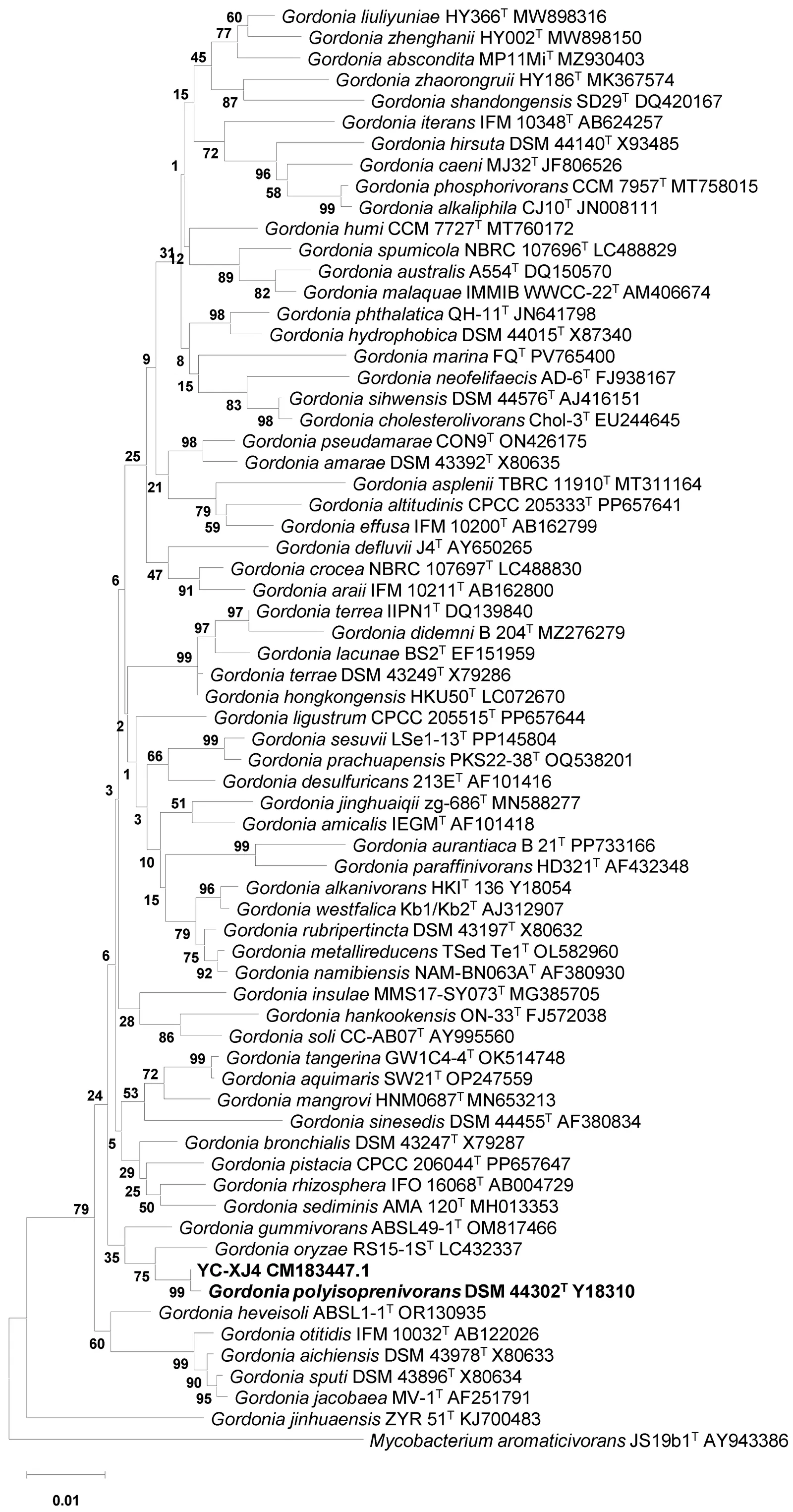
Phylogenetic tree based on 16S rDNA sequences. Neighbor-Joining [32] phylogenetic tree based on 68 16S rRNA gene sequences including 66 species of the genus *Gordonia* (including both validly published and Candidatus taxa), constructed using the Kimura 2-parameter model with gamma-distributed rates (shape = 1.00) [33]. Bootstrap support values (1000 replicates) are shown at the nodes [34]. Branch lengths are proportional to evolutionary distances (scale bar indicates substitutions per site). The final dataset comprised 1561 positions after pairwise deletion. The tree was generated with MEGA12 [35].

Whole-genome comparison showed that strain YC-XJ4 exhibited a dDDH value of 88.9% with the type strain of *Gordonia polyisoprenivorans*, which is well above the 70% species delineation threshold (Table S3) [27,28,29]. By contrast, dDDH values with all other *Gordonia* type strains ranged from 20.2% to 48.5%. Consistent with the dDDH result, the ANI values between strain YC-XJ4 and the type strains *G. polyisoprenivorans* ATCC BAA-14^T^ and NBRC 16320^T^ were 98.81% (GCA_012396285), 98.77% (GCA_000241325) and 98.80% (GCA_051303105) (Table S3), respectively, both well above the widely accepted 95–96% species delineation cutoff [31]. Phylogenetic analyses based on both the 16S rRNA gene sequence (Figure 2) and whole-genome data (phylogenomic tree, Figure S3) placed strain YC-XJ4 in the same clade as the type strain of *G. polyisoprenivorans*. Collectively, these phylogenetic data identify strain YC-XJ4 as *Gordonia polyisoprenivorans*.

Strain YC-XJ4 was deposited on 28 April 2026, at the China General Microbiological culture collection center (CGMCC, Institute of Microbiology, Chinese Academy of Sciences, No. 3, Yard 1, Beichen West Road, Chaoyang District, Beijing, China) under the accession number CGMCC No. 26483.

### 3.2. Characterization of TPHP Degradation Performance of Strain YC-XJ4

#### 3.2.1. Effects of Environmental Factors on TPHP Degradation by Strain YC-XJ4

The effects of temperature, pH, salinity, metal ions, and surfactants on the degradation of TPHP (200 mg/L) by strain YC-XJ4 were evaluated in TEM supplemented with TPHP as the sole carbon source.

**Figure 3:**
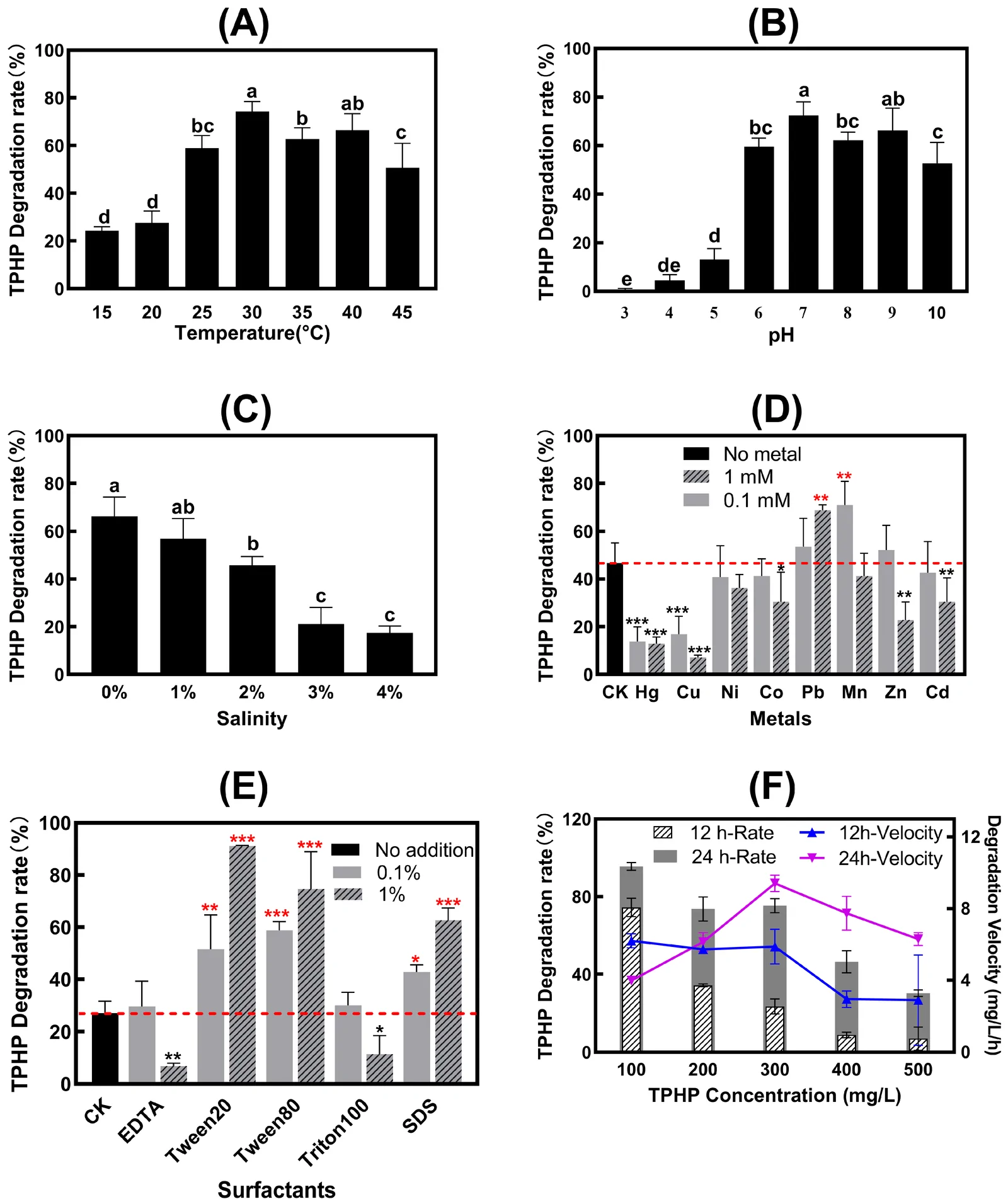
Effects of environmental factors on TPHP degradation by strain YC-XJ4. (**A**) Temperature; (**B**) pH; (**C**) salinity; (**D**) metal ions; (**E**) surfactants; (**F**) concentration gradient. In panels (**A**–**E**), unless otherwise indicated by the x-axis, cultures were incubated at pH 7.0 and 30 °C with an initial TPHP concentration of 200 mg/L. Incubation times were 24 h for (**A**–**C**), 16 h for (**D**), and 12 h for (**E**). Different lowercase letters above bars indicate significant differences (*p* < 0.05). Asterisks denote significance compared with CK (no metal or surfactant addition): * *p* < 0.05, ** *p* < 0.01, *** *p* < 0.001.

The optimum temperature for TPHP degradation was 30 °C, at which the degradation rate approached 80% after 24 h of incubation at pH 7.0. Notably, the strain retained over 20% degradation at temperatures as low as 15 °C and maintained more than 60% degradation even at 40 °C, demonstrating strong adaptability to temperature fluctuations (Figure 3A). At pH 6.0, the degradation rate was approximately 60%, and within the pH range of 8.0–10.0, the degradation rate remained above 50%, indicating robust degradation activity over a broad pH range (6.0–10.0) (Figure 3B).

Salinity effects were evaluated at pH 7.0 and 30 °C. With increasing NaCl concentrations (0–2%, *w*/*v*), strain YC-XJ4 maintained a degradation rate above 40% after 24 h of incubation, indicating moderate salt tolerance (Figure 3C). The effects of metal ions were examined at 30 °C and pH 7.0 after 16 h of incubation. Compared with the control without added metal ions, the presence of 0.1 mM Zn^2+^, Mn^2+^, and 1 mM Pb^2+^ stimulated TPHP degradation, whereas Hg^2+^ and Cu^2+^ (at both 0.1 and 1 mM) significantly inhibited degradation (Figure 3D). The effects of surfactants were assessed at 30 °C and pH 7.0 after 12 h of incubation. At a concentration of 0.1%, SDS, EDTA, Tween 20, Tween 80, and Triton X-100 all promoted TPHP degradation, with Tween 80 showing the most pronounced stimulatory effect. At 1%, Tween 20, Tween 80, and SDS still enhanced degradation, whereas EDTA and Triton X-100 substantially reduced the degradation rate (Figure 3E).

#### 3.2.2. Effect of Initial TPHP Concentration on Degradation by Strain YC-XJ4

The tolerance of strain YC-XJ4 to elevated TPHP concentrations was assessed in TEM with TPHP as the sole carbon source at 30 °C and pH 7.0. The initial TPHP concentration was varied from 100 to 500 mg/L at 100 mg/L intervals, and the degradation rate was determined after 12 h and 24 h of incubation. The degradation efficiency decreased progressively with increasing substrate concentration. Although the degradation rates declined, the average degradation velocities exhibited different patterns. After 12 h, the average degradation velocity decreased from 6.21 to 2.83 mg/(L·h) as the initial TPHP concentration increased from 100 to 500 mg/L. After 24 h, the average degradation velocity first increased and then decreased, peaking at 9.41 mg/(L·h) at an initial TPHP concentration of 300 mg/L. After 24 h, the degradation of 100 mg/L TPHP was nearly complete (95.57%), and even at the highest tested concentration of 500 mg/L, 30.23% of TPHP was still degraded by strain YC-XJ4, demonstrating its ability to tolerate and degrade relatively high levels of TPHP (Figure 3F).

#### 3.2.3. Degradation Capability of Strain YC-XJ4 Toward Different OPFRs

**Figure 4:**
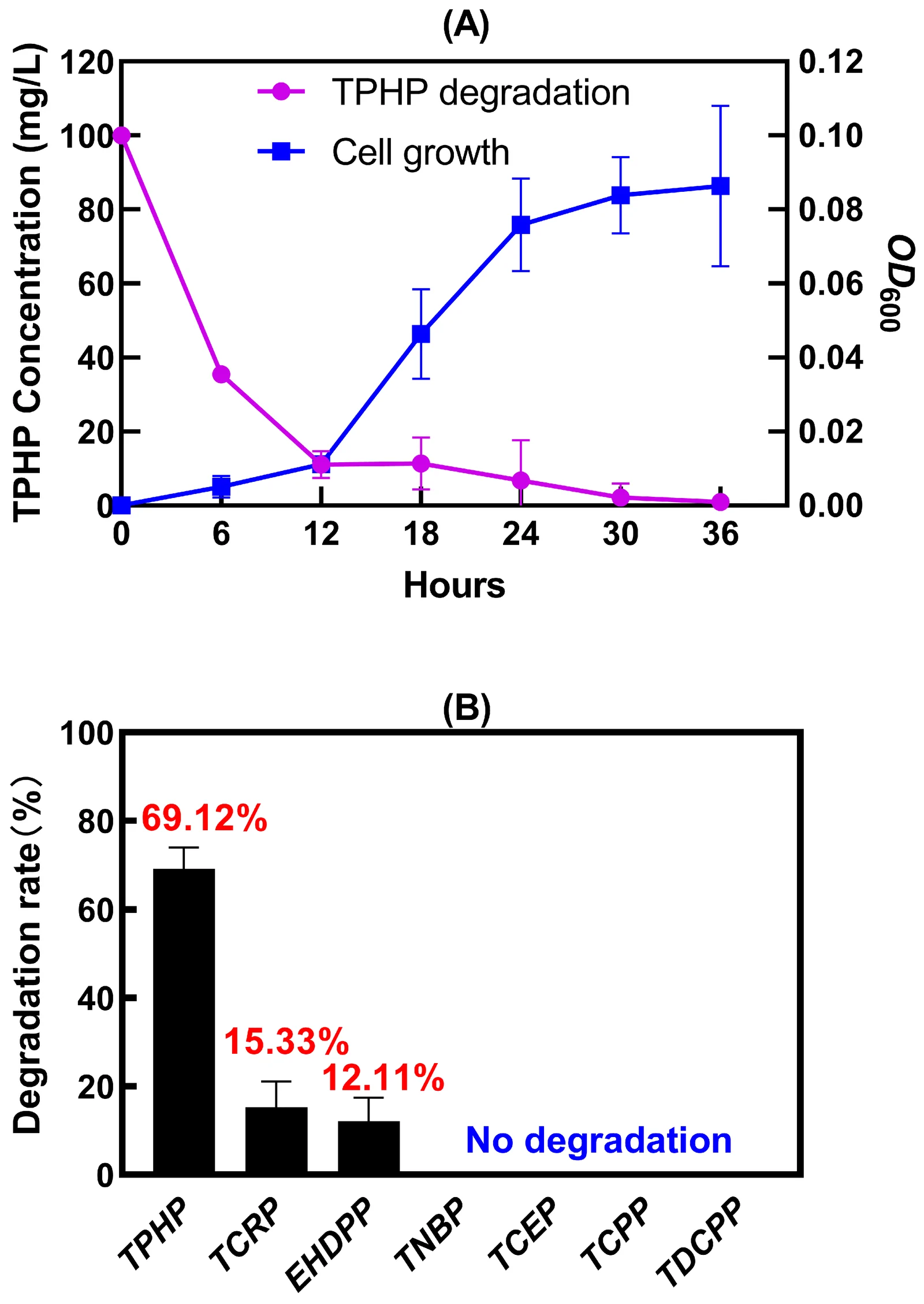
Degradation capability of strain YC-XJ4 toward different OPFRs. (**A**) Degradation curve of TPHP (100 mg/L) and growth curve of the strain (pH 7.0, 30 °C); (**B**) substrate spectrum of OPFRs (initial concentration, 200 mg/L) degradation by strain YC-XJ4 (pH 7.0, 30 °C, 24 h).

The degradation kinetics and cell growth of strain YC-XJ4 on TPHP were evaluated under optimal conditions (30 °C, pH 7.0) with an initial TPHP concentration of 100 mg/L. Approximately 90% of TPHP was degraded within 12 h of incubation, and degradation reached nearly 99% as the incubation was extended to 36 h. Apparent cell growth was observed after 12 h, with the *OD*_600_ value increasing rapidly and then stabilizing after 24 h; after 36 h, the *OD*_600_ exceeded 0.08 (Figure 4A).

The substrate specificity of strain YC-XJ4 was determined at 30 °C and pH 7.0 using various OPFRs (TPHP, TCRP, EHDPP, TNBP, TCEP, TCPP, TDCPP) at an initial concentration of 200 mg/L. After 24 h of incubation, in addition to TPHP, the strain also degraded TCRP and EHDPP. Like TPHP, both TCRP and EHDPP belong to the aryl-OPFRs. However, the degradation rate of TPHP (nearly 70%) was much higher than those of TCRP and EHDPP, which were only in the range of 10–20%. Under the tested conditions, no degradation of TNBP, TCEP, TCPP, or TDCPP was detected (Figure 4B).

### 3.3. Assessment of the Bioremediation Potential for TPHP Pollution in Soil and Seawater

**Figure 5:**
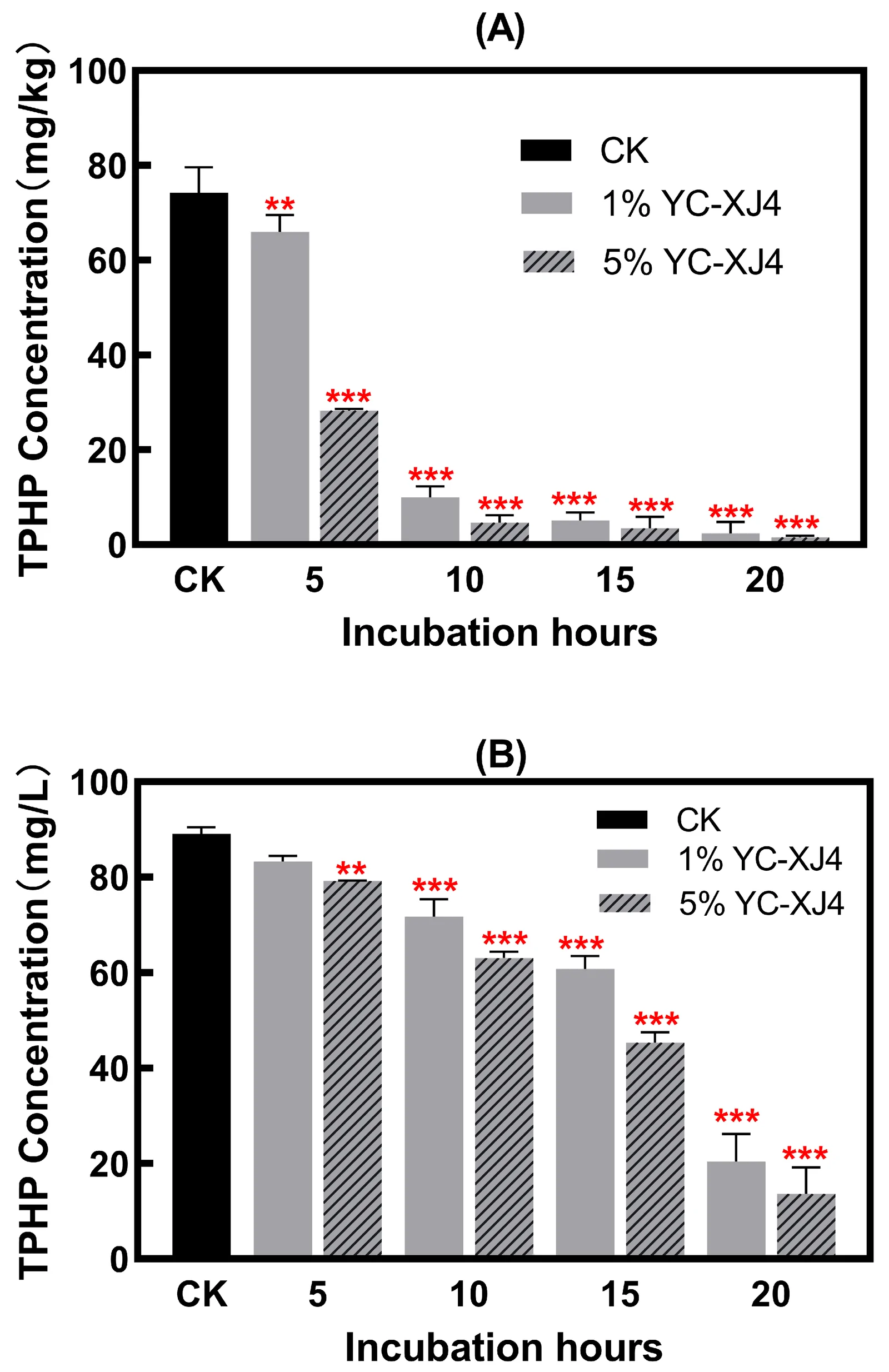
Application of *G. polyisoprenivorans* YC-XJ4 in the bioremediation of contaminated environments. (**A**) Soil; (**B**) seawater. Soil was spiked with TPHP at 100 mg/kg, and seawater was spiked with TPHP at 100 mg/L. CK, uninoculated soil or seawater; 1% YC-XJ4, treatment inoculated with 1% (*v*/*w* in soil, *v*/*v* in seawater) YC-XJ4; 5% YC-XJ4, treatment inoculated with 5% YC-XJ4. Asterisks denote significance compared with CK (uninoculated): ** *p* < 0.01, *** *p* < 0.001.

The potential of strain YC-XJ4 to remediate TPHP contamination was evaluated in both soil and seawater under natural outdoor conditions. For soil bioremediation, garden soil was artificially spiked with 100 mg/kg TPHP, receiving 1% or 5% (*v*/*v*) inoculum of YC-XJ4. After 20 h of incubation, the residual TPHP concentration in the uninoculated control decreased from the initial 100 mg/kg to 74.22 mg/kg (25.78% removal), indicating that indigenous soil microorganisms contributed partially to TPHP removal. In the inoculated groups, the residual TPHP concentrations were consistently lower than those in the control. With a 5% inoculum, the TPHP concentration declined sharply to 28.30 mg/kg within the first 5 h (71.7% removal) and 4.66 mg/kg within 10 h (95.34% removal), demonstrating a rapid and efficient removal by strain YC-XJ4. With a 1% inoculum, the residual TPHP concentration decreased only slightly to 65.95 mg/kg after 5 h (34.05% removal), but dropped markedly to 9.95 mg/kg after 10 h (90.05% removal), indicating a lag phase followed by accelerated removal. Overall, although native soil microbes exhibited some TPHP-removing activity, the inoculation of YC-XJ4 significantly enhanced and accelerated the removal of TPHP from the soil, and increasing the inoculum size from 1% to 5% markedly improved the remediation efficiency (Figure 5A).

For seawater bioremediation, coastal seawater was spiked with 100 mg/L TPHP and inoculated with 1% or 5% (*v*/*v*) YC-XJ4 suspension, with uninoculated and unsterilized seawater containing the same TPHP concentration serving as the control. Over the 20 h incubation period, the residual TPHP concentration decreased progressively in all inoculated treatments. Both 1% and 5% inoculum sizes effectively reduced TPHP levels, but the 5% inoculation led to a greater extent of removal at each time point. At 20 h, the removal efficiency reached 79.53% with 1% inoculum and 86.41% with 5% inoculum, while only 10.99% of TPHP was removed in the uninoculated and unsterilized control. These results indicate that the indigenous microorganisms in seawater could partially remove TPHP, whereas the addition of YC-XJ4 exhibited superior TPHP removal capacity. At the same sampling time points, the higher inoculum density (5%) consistently resulted in faster TPHP removal than the lower inoculum density (1%), demonstrating the strain’s pronounced potential for bioremediation of TPHP contaminated seawater (Figure 5B).

## 4. Discussion

In this study, a highly efficient TPHP-degrading bacterium, *G. polyisoprenivorans* YC-XJ4, was isolated from plastic-waste-contaminated soil. The strain demonstrated robust degradation capability over a wide range of temperature (15–40 °C), pH (6–10), and salinity (0–2% NaCl), with optimal activity at 30 °C and pH 7.0. Notably, YC-XJ4 tolerated TPHP concentrations up to 500 mg/L and achieved a maximum average degradation velocity of 9.41 mg/(L·h) at 300 mg/L. The strain preferentially degraded aryl-OPFRs (TPHP, TCRP, EHDPP) but not chlorinated or alkyl congeners. Importantly, YC-XJ4 exhibited excellent TPHP removal in simulated soil and seawater bioremediation experiments, with enhanced performance at higher inoculum densities. As a non-*Sphingomonadaceae* degrader with exceptional environmental adaptability and proven bioremediation potential, YC-XJ4 represents a valuable candidate for the bioremediation of OPFR-contaminated environments.

To date, a considerable number of bacterial strains capable of degrading TPHP and other OPFRs have been isolated from various environmental niches (Table 1). Based on their average degradation velocities (calculated from reported degradation rates), these isolates can be broadly divided into two categories: highly efficient degraders (average velocity > 1 mg/(L·h)) and weak degraders (average velocity ≤ 600 μg/(L·h)). Notably, the majority of highly efficient TPHP-degrading strains belong to the family *Sphingomonadaceae*, encompassing the genera *Sphingomonas*, *Sphingobium*, and *Sphingopyxis*. Representative examples include *Sphingobium yanoikuyae* YC-XJ2 (4.12 mg/(L·h)) [16], *Sphingopyxis terrae* subsp. *terrae* YC-JH3 (3.65 mg/(L·h)) [18,36], and *Sphingobium* sp. RSMS (110.83 mg/(L·h) for TNBP, though its TPHP velocity is not reported [14]. The exceptional degradation efficiency of these *Sphingomonadaceae* strains could be attributed to the presence of OPFR-specific phosphotriesterases (PTEs), including *Sm*-PTE, *Sb*-PTE, *Sy*-PTE, and *St*-PTE, which hydrolyze TPHP to diphenyl phosphate (DPHP) with high catalytic efficiency [13,16,36,37,38]. Notably, these four PTEs share over 90% nucleotide sequence identity and are highly conserved, yet they have been found exclusively within the *Sphingomonadaceae* family, suggesting a lineage-specific evolutionary origin of this efficient degradation mechanism [36].

However, several non-*Sphingomonadaceae* strains have also demonstrated remarkable TPHP degradation. For instance, *Rhodococcus pyridinivorans* YC-MTN achieved an average velocity of 4.01 mg/(L·h) for TPHP [22], and *Stutzerimonas frequens* RL-XB02 exhibited a velocity of approximately 2.08 mg/(L·h) [42]. Intriguingly, genome analysis of *R. pyridinivorans* YC-JH2, another *Rhodococcus* strain YC-JH2 with moderate TPHP-degrading ability (111.19 μg/(L·h)), revealed the absence of any OPFR-specific PTE homologs [17], indicating that an entirely different, yet-to-be-elucidated enzymatic mechanism underlies its degradation capacity. Unfortunately, the whole-genome sequence of *S. frequens* RL-XB02 is not yet publicly available in GenBank or other databases; consequently, its PTE status cannot be determined, and its degradation mechanism remains unresolved. In contrast, many other reported degraders, such as *Brevibacillus brevis* (7.68 μg/(L·h)) [20,21] and *Roseobacter* strain YS-57 (23.33 μg/(L·h)) [19], exhibit substantially lower degradation efficiencies.

Strain YC-XJ4, identified as *Gordonia polyisoprenivorans*, displayed an average TPHP degradation velocity of 9.41 mg/(L·h) (at an initial concentration of 300 mg/L), which places it firmly within the group of highly efficient degraders. This value is notably higher than those of most previously reported non-*Sphingomonadaceae* degraders, including *R. pyridinivorans* YC-MTN (4.01 mg/(L·h)) and *S. frequens* RL-XB02 (2.08 mg/(L·h)), and is comparable to or even exceeds some *Sphingomonadaceae* members (e.g., YC-XJ2 and YC-JH3). Importantly, homologous sequence analysis of the YC-XJ4 genome revealed no OPFR-specific PTE-encoding genes (Figure S4), suggesting that its high-efficiency TPHP degradation is mediated by a novel enzymatic pathway distinct from the well-characterized PTE system of the *Sphingomonadaceae*. In addition to the well-known PTEs predominantly found in the *Sphingomonadaceae*, several non-PTE enzymes have been functionally characterized in *Gordonia* species that could potentially act on OPFRs. For instance, several *Gordonia* strains harbor hydrolases and oxidoreductases capable of cleaving ester bonds and modifying aromatic rings. In *Gordonia* sp. YC-JH1 and *Gordonia alkanivorans* YC-RL2, genes encoding di-esterases and mono-esterases have been annotated, which can sequentially hydrolyze the two ester bonds of phthalate diesters to generate phthalic acid [43,44,45]. Similarly, a mono-alkyl phthalate hydrolase was identified in *Gordonia* sp. LUNF6 [46]. Furthermore, the genomes of *Gordonia polyisoprenivorans* B251 and B253 contain genes encoding oxidoreductases, hydrolases, and monooxygenases that target ester bonds, aromatic rings, or carbon–carbon double bonds in polymer backbones [26]. The presence of such enzymes in *Gordonia* is particularly relevant to our strain YC-XJ4, which lacks the annotated OPFR-specific PTE genes but exhibits efficient degradation of TPHP and other aryl-OPFRs. Therefore, it is reasonable to hypothesize that YC-XJ4 employs one or more of these non-PTE enzymes to initiate OPFR degradation, and future enzymatic studies will be directed toward identifying the metabolic intermediates and the specific enzymes involved. Thus, along with *R. pyridinivorans* YC-JH2 and *S. frequens* RL-XB02 (whose PTE status remains indeterminate due to the unavailability of genomic data), YC-XJ4 represents another case of a highly efficient TPHP degrader whose molecular mechanism remains unknown, highlighting the existence of diverse, unexplored catalytic strategies for OPFR breakdown beyond the known PTE paradigm. Future studies should therefore focus on identifying the key enzyme(s) responsible for TPHP hydrolysis in YC-XJ4 through genomic mining, transcriptomic profiling, or proteomic approaches.

Notably, the degradation velocity of YC-XJ4 was concentration-dependent: the maximum average velocity was achieved at 300 mg/L, whereas higher or lower substrate concentrations resulted in reduced velocities, suggesting that substrate inhibition or growth limitation occurs at elevated TPHP levels. Although the degradation curve of TPHP (100 mg/L) could be fitted by a pseudo-first-order kinetic model, the derived rate constant (*k* = 0.127 h^−1^) was not universally applicable across different initial concentrations, as the calculated degradation rates at higher TPHP levels deviated substantially from actual values. This discrepancy indicates that elevated TPHP concentrations suppress the growth and metabolic activity of strain YC-XJ4, which aligns with the observed reduction in degradation efficiency at concentrations above 300 mg/L. Nevertheless, even at 500 mg/L, YC-XJ4 retained approximately 30% degradation activity, highlighting its remarkable tolerance to high substrate loads. Furthermore, YC-XJ4 exhibited a preferential substrate spectrum toward aryl-OPFRs (TPHP, TCRP, and EHDPP), with no detectable degradation of chlorinated (TCEP, TCPP, TDCPP) or alkyl (TNBP) congeners under the tested conditions, a pattern similar to that of many *Sphingomonadaceae* strains but distinct from broader-spectrum degraders such as *B. pacificus* L2, capable of degrading TCEP and TCPP in addition to TPHP and TCRP [41].

Collectively, these comparisons demonstrate that *G. polyisoprenivorans* YC-XJ4 represents a rare and valuable non-*Sphingomonadaceae* degrader with competitive degradation kinetics, a distinct substrate preference, and its degradation mechanism likely involves novel enzymes that remain to be identified, thereby enriching the repertoire of microbial resources for OPFR bioremediation and providing a promising starting point for enzyme discovery.

Although strain YC-XJ4 efficiently removed TPHP from both culture medium and contaminated environmental matrices, the present study primarily monitored the disappearance of the parent compound and did not identify the degradation intermediates or verify complete mineralization. Therefore, the observed decrease in TPHP concentration should be interpreted as TPHP removal rather than complete biodegradation. To fully elucidate the metabolic fate of TPHP, future work should focus on identifying the intermediates generated during degradation using techniques such as liquid chromatography–tandem mass spectrometry (LC-MS/MS) and gas chromatography–mass spectrometry (GC-MS), and on evaluating whether these metabolites are further mineralized to CO_2_ and H_2_O. In addition, the enzymatic basis of the high-efficiency TPHP degradation observed in strain YC-XJ4 remains to be clarified. The construction of a genomic library, heterologous expression of candidate genes, and more detailed genome functional annotation and comparative genomic analyses will be essential for identifying the specific enzymes responsible for TPHP hydrolysis and for understanding the underlying metabolic mechanism. These efforts will not only improve our understanding of the degradation pathway but also facilitate the safe and efficient application of strain YC-XJ4 in bioremediation.

## 5. Conclusions

This study successfully isolated and characterized a highly efficient TPHP-degrading bacterium, *G. polyisoprenivorans* YC-XJ4, from plastic-waste-contaminated soil. Strain YC-XJ4 exhibited excellent degradation performance across a broad range of environmental conditions, including temperatures from 15 °C to 40 °C, pH values from 6.0 to 10.0, and salinities up to 2% (*w*/*v*) NaCl, demonstrating remarkable environmental adaptability. The strain achieved a maximum average degradation velocity of 9.41 mg/(L·h) at an initial TPHP concentration of 300 mg/L and retained approximately 30% degradation activity even at 500 mg/L, underscoring its strong tolerance to high substrate loads. Substrate spectrum analysis revealed a preferential activity toward aryl-OPFRs (TPHP, TCRP, and EHDPP), with no detectable degradation of chlorinated or alkyl congeners. Importantly, whole-genome sequencing and homologous sequence analysis confirmed the absence of OPFR-specific phosphotriesterase genes in YC-XJ4, suggesting that its high-efficiency TPHP degradation may be mediated by novel enzymes distinct from the well-characterized system in *Sphingomonadaceae* strains. Furthermore, simulated bioremediation experiments demonstrated that YC-XJ4 effectively removed TPHP from both soil and seawater under natural outdoor conditions, with higher inoculum densities (5%, *v*/*v*) achieving over 95% removal in soil within 10 h and over 84% removal in seawater within 20 h. Collectively, these findings position *G. polyisoprenivorans* YC-XJ4 as a rare and valuable non-*Sphingomonadaceae* degrader with competitive degradation capacity, broad environmental tolerance, proven bioremediation potential, and a yet-to-be-elucidated degradation mechanism. This study not only enriches the microbial resource pool for OPFR bioremediation but also provides a promising candidate for practical application in contaminated environments. Future work should focus on elucidating the key enzyme(s) and metabolic pathway responsible for TPHP hydrolysis in YC-XJ4 through genomic mining, transcriptomic profiling, and proteomic approaches, which may uncover novel biocatalysts for the efficient removal of organophosphate pollutants.

## Figures and Tables

**Table 1:** Bacterial strains capable of degrading OPFRs.

Strains	Ability to Degrade OPFRs	Phosphotriesterase ^a^	Reference
*Sphingomonas* sp. TDK1	TDCPP (1.08 mg/(L·h))	*Sm*-PTE	[13,38]
*Sphingobium* sp. TCM1	TCEP (1.90 mg/(L·h)), TDCPP (1.43 mg/(L·h))	*Sb*-PTE (*k*_cat_/*K*_m_ of TPHP: 1.7 × 10^6^ 1/(M·s))	[13,37,38]
*Sphingobium* sp. RSMS	TNBP (110.83 mg/(L·h))	-	[14]
*Sphingomonas* sp.	TNBP (1.43 mg/(L·h))	-	[15]
*Sphingobium yanoikuyae* YC-XJ2	TPHP (4.12 mg/(L·h))	*Sy*-PTE (*k*_cat_/*K*_m_ of TPHP: 4.8 × 10^6^ 1/(M·s))	[16]
*Sphingopyxis terrae* subsp. *terrae* YC-JH3	TPHP (3.65 mg/(L·h))	*St*-PTE (*k*_cat_/*K*_m_ of TPHP: 5.03 × 10^6^ 1/(M·s))	[17,18,36]
*Sphingopyxis* sp. GY	TPHP (41.67 μg/(L·h))	-	[39]
*Sphingobium yanoikuyae* YC-JY1	TPHP (573.61 μg/(L·h))	N	[40]
*Roseobacter* strain YS-57	TPHP (23.33 μg/(L·h)), TCRP (8.75 μg/(L·h))	-	[19]
*Brevibacillus brevis*	TPHP (7.68 μg/(L·h)), *m*-TCRP (6.91 μg/(L·h)), *p*-TCRP (7.82 μg/(L·h)), *o*-TCRP (4.50 μg/(L·h))	N	[20,21]
*Rhodococcus pyridinivorans* YC-JH2	TPHP (111.19 μg/(L·h))	N	[17,18]
*Rhodococcus pyridinivorans* YC-MTN	TPHP (4.01 mg/(L·h))	N	[22]
*Bacillus pacificus* L2	TCRP (≈44 μg/(L·h)) TPHP (≈31 μg/(L·h)) TCPP (≈6.3 μg/(L·h)) TCEP (≈5.2 μg/(L·h))	-	[41]
*Stutzerimonas frequens* RL-XB02	TPHP (≈2.08 mg/(L·h))	-	[42]
*Gordonia polyisoprenivorans* YC-XJ4	TPHP (9.41 mg/(L·h))	N	This study

Note: ^a^ Whether the strain has the specific OPFRs hydrolyzing phosphotriesterase; “-” indicates that there is no research on this topic; N means no OPFR-specific phosphotriesterase can be found.

## Data Availability

The original contributions presented in this study are included in the article/Supplementary Materials. Further inquiries can be directed to the corresponding authors.

## Supplementary Materials

The following supporting information can be downloaded at https://www.mdpi.com/article/10.3390/toxics14090815/s1, Table S1: Experimental design of soil bioremediation with strain YC-XJ4; Table S2: Experimental design of seawater bioremediation with strain YC-XJ4; Table S3: Genomic pairwise comparisons of the strain YC-XJ4 vs. the closest *Gordonia* type strains; Figure S1: Standard curve for the detection of OPFRs (TPHP, TCRP and EHDPP) by HPLC; Figure S2: Standard curve for the detection of OPFRs (TNBP, TCPP, TCEP and TDCPP) by GC; Figure S3: Whole genome based phylogenomic tree constructed by based on the Type (Strain) Genome Server (TYGS); Figure S4: The sequence alignment results of the homologous regions between *Sb*-PTE and the genome of strain YC-XJ4. References [27,28,29,30] are cited in Supplementary Materials.
