# Supplementary material for: Biodegradation of Triphenyl Phosphate by Gordonia polyisoprenivorans YC-XJ4: Environmental Adaptability and Bioremediation Potential
Source: Toxics. 2026 Sep 13;14(9):815. doi: 10.3390/toxics14090815 (PMC13611039; doi:10.3390/toxics14090815)
Supplement: Supplementary file 1 [file toxics-14-00815-s001.zip › toxics-4512842-supplementary.pdf]

Biodegradation of Triphenyl Phosphate by *Gordonia polyisoprenivorans* YC-XJ4: Environmental Adaptability and Bioremediation Potential

Xianjun Li [1], Xiao Ye [1], Junhuan Wang [2,*], Aikebaier Reheman [1,*]

[1] Fujian Key Laboratory of Toxicant and Drug Toxicology, Medical College, Ningde Normal University, Ningde, 352100, China; lixianjun1978@126.com (X. L.); yexiao_817@163.com (X. Y.)

[2] School of Ocean Food and Biological Engineering, Jiangsu Ocean University, Lianyungang, Jiangsu, 222005, China

* Correspondence: 2025000084@jou.edu.cn (J. W.); akbarphd@126.com (A. R.);

Table S1 Experimental design of soil bioremediation with strain YC-XJ4

Groups	inoculation (v/v)	Inoculum of YC-XJ4 (mL)	Final concentration of TPHP (mg/kg)	TEM (mL)
CK (Unsterilized soil)	0	0	100	5
1% (Sterilized soil)	1%	0.1	100	4.9
5% (Sterilized soil)	5%	0.5	100	4.5

Table S2 Experimental design of seawater bioremediation with strain YC-XJ4

Group	Inoculum of YC-XJ4 (μL)	TPHP stock solution (μL)	Final concentration of TPHP (mg/L)	Seawater (mL)
CK	0	50	100	10 (Unfiltered)
1%	100	50	100	9.9 (Filtered)
5%	500	50	100	9.5 (Filtered)

Table S3 Genomic pairwise comparisons of the strain YC-XJ4 vs. the closest *Gordonia* type strains

Subject strain	Digital DNA–DNA hybridization (dDDH) (%) [a]	Average Nucleotide Identity (ANI) [b]	Assembly accession [c]
G. polyisoprenivorans ATCC BAA-14	**88.9**	**98.81**	GCA_012396285
G. polyisoprenivorans NBRC 16320	**88.9**	**98.77**	GCA_000241325
G. polyisoprenivorans NBRC 16320	**88.9**	**98.80**	GCA_051303105
G. oryzae RS15-1S	48.5	92.79	GCA_003797185
G. westfalica DSM 44215	22.2	76.42	GCA_900105725
G. rubripertincta NBRC 101908	21.8	76.55	GCA_000327325
G. aquimaris SW21	21.8	76.37	GCA_026262595
G. desulfuricans NBRC 100010	21.6	77.94	GCA_001485495
G. desulfuricans 213E	21.6	78.11	GCA_010119475
G. bronchialis NCTC 10667	21.1	76.25	GCA_900450805
G. aurantiaca B21	21.1	76.42	GCA_039939235
G. bronchialis DSM 43247	21	76.18	GCA_000024785
G. rhizosphera NBRC 16068	20.9	76.08	GCA_000298195

G. gummivorans ABSL49-1	20.9	75.9	GCA_022513585
G. prachuapensis PKS22-38	20.5	75.55	GCF_036350515
G. insulae MMS17-SY073T	20.3	75.62	GCF_003855095
G. soli NBRC 108243	20.2	75.41	GCA_000334455

Note: [a] dDDH values were calculated using formula d_4 via the Type (Strain) Genome Server (TYGS) (Freese et al., 2026 , Meier-Kolthoff et al., 2022 , Meier-Kolthoff and Göker, 2019), with the genome sequence of strain YC-XJ4 (GenBank accession GCA_059511865.1) used as the query against all type strain genomes listed. [b] ANI values were calculated using online calculator (https://www.ezbiocloud.net/tools/ani) (Yoon et al., 2017). [c] Assembly accession numbers for the subject genomes are provided.

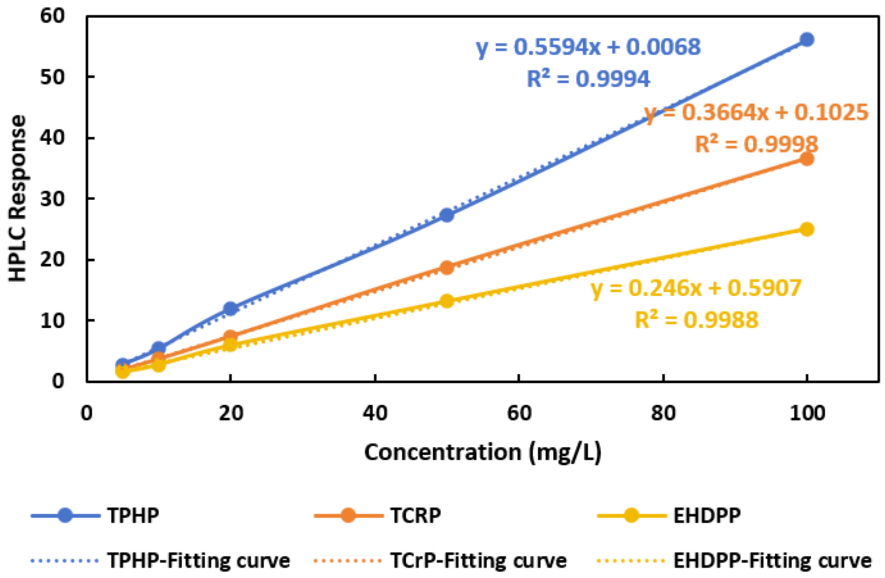

Figure S1 Standard curve for the detection of OPFRs (TPHP, TCRP and EHDPP) by HPLC

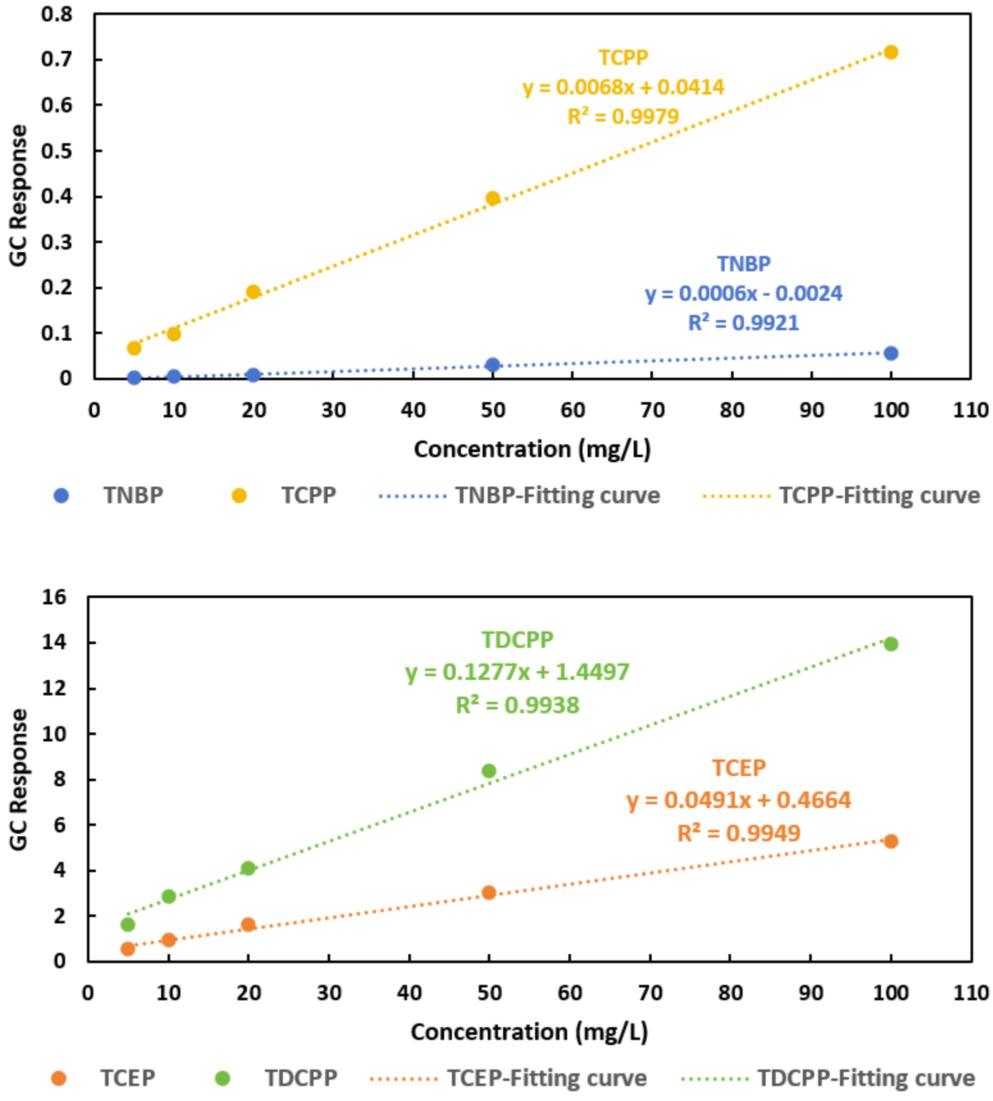

Figure S2 Standard curve for the detection of OPFRs (TNBP, TCPP, TCEP and TDCPP) by GC

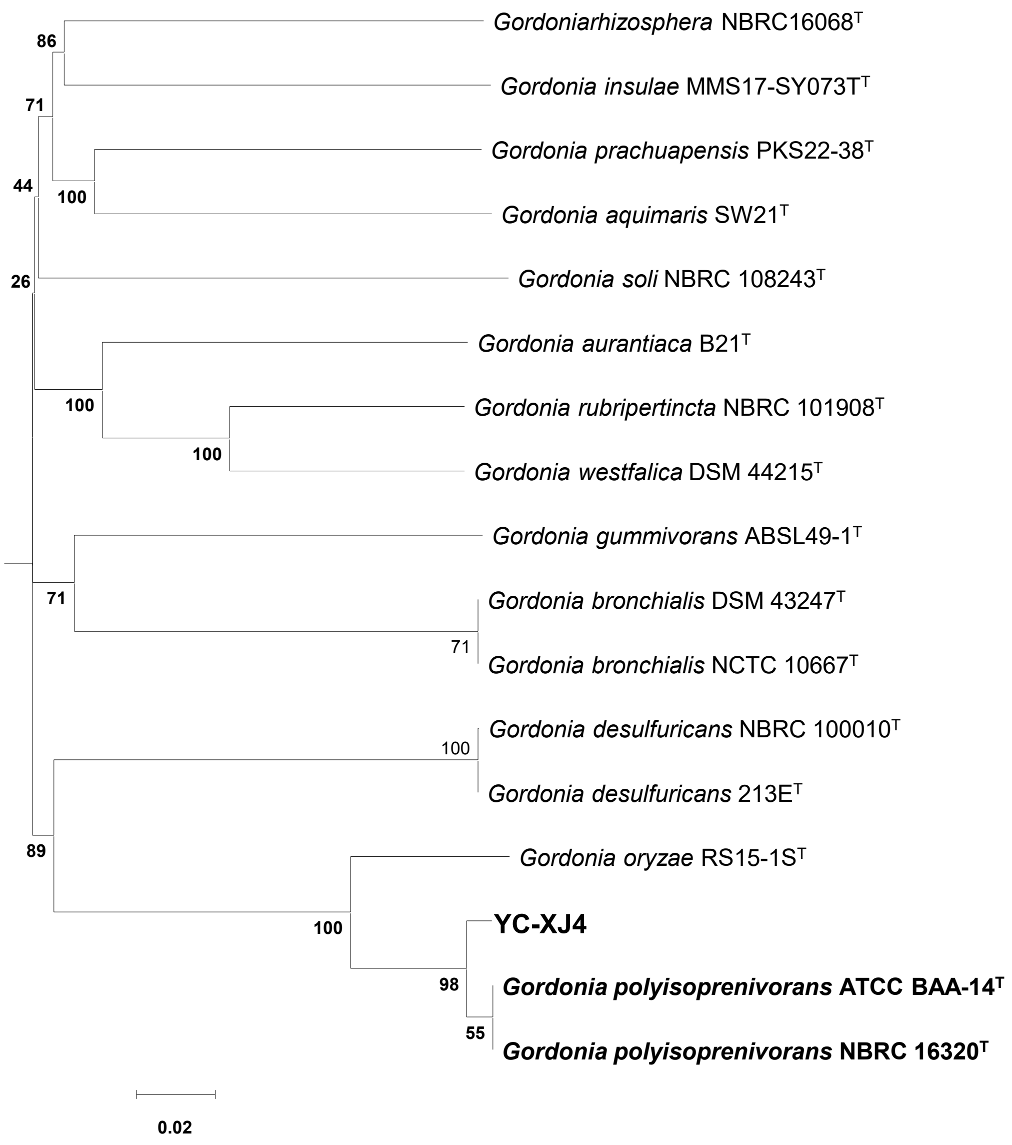

Figure S3 Whole genome based phylogenomic tree constructed by based on the Type (Strain) Genome Server (TYGS)

The accession numbers of these genomes were listed in Table S3

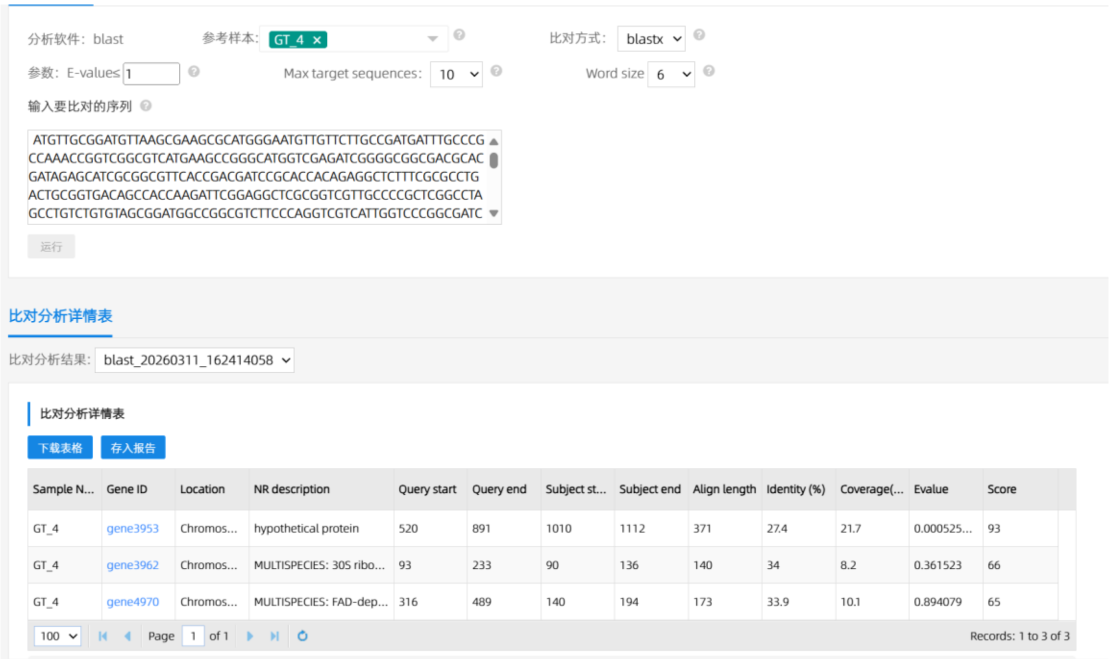

Sample N...	Gene ID	Location	NR description	Query start	Query end	Subject st...	Subject end	Align length	Identity (%)	Coverage(...	Evalue	Score
GT_4	gene3953	Chromos...	hypothetical protein	520	891	1010	1112	371	27.4	21.7	0.000525...	93
GT_4	gene3962	Chromos...	MULTISPECIES: 30S ribo...	93	233	90	136	140	34	8.2	0.361523	66
GT_4	gene4970	Chromos...	MULTISPECIES: FAD-dep...	316	489	140	194	173	33.9	10.1	0.894079	65

Figure S4 The sequence alignment results of the homologous regions between *Sb*-PTE and the genome of strain YC-XJ4

References

FREESE H M, MEIER-KOLTHOFF J P, SARDà CARBASSE J, et al. 2026. TYGS and LPSN in 2025: a Global Core Biodata Resource for genome-based classification and nomenclature of prokaryotes within DSMZ Digital Diversity. Nucleic Acids Research [J], 54: D884-D891.

MEIER-KOLTHOFF J P, CARBASSE J S, PEINADO-OLARTE R L, et al. 2022. TYGS and LPSN: a database tandem for fast and reliable genome-based classification and nomenclature of prokaryotes. Nucleic Acids Research [J], 50: D801-D807.

MEIER-KOLTHOFF J P, GöKER M 2019. TYGS is an automated high-throughput platform for state-of-the-art genome-based taxonomy. Nature Communications [J], 10: 2182.

YOON S-H, HA S-M, LIM J, et al. 2017. A large-scale evaluation of algorithms to calculate average nucleotide identity. Antonie van Leeuwenhoek [J], 110: 1281-1286.
